# TREM2 deficiency reduces the efficacy of immunotherapeutic amyloid clearance

**DOI:** 10.15252/emmm.201606370

**Published:** 2016-07-08

**Authors:** Xianyuan Xiang, Georg Werner, Bernd Bohrmann, Arthur Liesz, Fargol Mazaheri, Anja Capell, Regina Feederle, Irene Knuesel, Gernot Kleinberger, Christian Haass

**Affiliations:** ^1^ Biomedical Center (BMC)BiochemistryLudwig‐Maximilians‐University MunichMunichGermany; ^2^ Graduate School of Systemic NeuroscienceLudwig‐Maximilians‐University MunichMunichGermany; ^3^ Roche Pharmaceutical Research and Early Development NORD Discovery & Translational AreaRoche Innovation Center BaselBaselSwitzerland; ^4^ Institute for Stroke and Dementia ResearchKlinikum der Universität MünchenMunichGermany; ^5^ Munich Cluster for Systems Neurology (SyNergy)MunichGermany; ^6^ German Center for Neurodegenerative Diseases (DZNE) MunichMunichGermany; ^7^ Helmholtz Center MunichGerman Research Center for Environmental HealthInstitute for Diabetes and ObesityCore Facility Monoclonal Antibody DevelopmentMunichGermany

**Keywords:** Alzheimer's disease, immunotherapy, neurodegeneration, phagocytosis, TREM2, Immunology, Neuroscience, Pharmacology & Drug Discovery

## Abstract

Immunotherapeutic approaches are currently the most advanced treatments for Alzheimer's disease (AD). Antibodies against amyloid β‐peptide (Aβ) bind to amyloid plaques and induce their clearance by microglia via Fc receptor‐mediated phagocytosis. Dysfunctions of microglia may play a pivotal role in AD pathogenesis and could result in reduced efficacy of antibody‐mediated Aβ clearance. Recently, heterozygous mutations in the triggering receptor expressed on myeloid cells 2 (*TREM2*), a microglial gene involved in phagocytosis, were genetically linked to late onset AD. Loss of TREM2 reduces the ability of microglia to engulf Aβ. We have now investigated whether loss of TREM2 affects the efficacy of immunotherapeutic approaches. We show that anti‐Aβ antibodies stimulate Aβ uptake and amyloid plaque clearance in a dose‐dependent manner in the presence or absence of TREM2. However, TREM2‐deficient N9 microglial cell lines, macrophages as well as primary microglia showed significantly reduced uptake of antibody‐bound Aβ and as a consequence reduced clearance of amyloid plaques. Titration experiments revealed that reduced efficacy of amyloid plaque clearance by *Trem2* knockout cells can be compensated by elevating the concentration of therapeutic antibodies.

## Introduction

Alzheimer's disease (AD) is the most abundant neurodegenerative disorder and threatens our aging society. Therapeutic treatment is desperately required to slow progression of dementia. The amyloid cascade (Hardy & Selkoe, 2002) provides a number of opportunities to therapeutically interfere with disease onset and progression. Obvious targets are β‐ and γ‐secretases, the two proteases, which generate the amyloid β‐peptide (Aβ) from its precursor, the β‐amyloid precursor protein (APP) (Haass, 2004). γ‐Secretase inhibition caused major side effects in a clinical trial, which were at least partially due to inhibition of its biological activity in Notch signaling (Doody *et al*, 2013). Similarly, recent findings may also indicate that inhibition of β‐secretase may be problematic due to the accumulation of alternative neurotoxic APP‐derived fragments (Willem *et al*, 2015). Moreover, β‐secretase has numerous brain‐specific substrates and their biological function may be disturbed upon inhibition of shedding (Kuhn *et al*, 2012; Zhou *et al*, 2012; Barao *et al*, 2016). In fact, neuregulin‐1 signaling depends on biologically active β‐secretase (Willem *et al*, 2006; Cheret *et al*, 2013). Nevertheless, several β‐secretase inhibitors are currently in late‐stage clinical development and if demonstrated to be safe may be of therapeutic value. The most advanced and very promising therapeutic approach is currently the anti‐Aβ immunotherapy (Wisniewski & Goni, 2015), a treatment strategy initiated after the pivotal report by Schenk *et al* (Schenk *et al*, 1999) demonstrating its efficacy in amyloid plaque removal and reduction in memory decline in an animal model (Morgan *et al*, 2000). After crossing the blood–brain barrier, anti‐Aβ antibodies like 3D6 and others bind to amyloid plaques and trigger Fc receptor‐mediated phagocytosis of plaques by microglia cells (Bard *et al*, 2000). Indeed, 11C‐PiB positron emission tomography (PET) revealed a significant reduction in amyloid deposition in patients treated with anti‐Aβ antibodies (Rinne *et al*, 2010; Ostrowitzki *et al*, 2012). Although initially there was no obvious beneficial effect on memory, data from two recent clinical trials now suggest that immunotherapy may significantly slow memory decline (Reardon, 2015). Moreover, a recent clinical trial using Aducanumab indicated that significant amyloid removal is required for clinical benefits suggesting a critical role of amyloid removal by microglia. Indeed, antibody‐mediated clearance of amyloid plaques requires biologically active microglia cells (Bard *et al*, 2000). Recently, it was reported that heterozygous mutations in the triggering receptor expressed on myeloid cells 2 (*TREM2*) increase the risk for AD, frontotemporal lobar degeneration, amyotrophic lateral sclerosis, and Parkinson's disease (Guerreiro *et al*, 2013; Jonsson *et al*, 2013; Rayaprolu *et al*, 2013; Borroni *et al*, 2014; Cady *et al*, 2014; Cuyvers *et al*, 2014; Colonna & Wang, 2016; Ulrich & Holtzman, 2016; Villegas‐Llerena *et al*, 2016). We have shown that certain mutations in TREM2 such as p.T66M reduce the ability of microglia to phagocytose Aβ fibers, bacteria, and beads due to impairment of TREM2 maturation and cell surface transport (Kleinberger *et al*, 2014). Furthermore, a complete knockout of TREM2 also reduces the ability of primary microglia to phagocytose Aβ (Kleinberger *et al*, 2014). Similarly, in a cuprizone model for acute demyelination, TREM2‐deficient mice showed reduced phagocytic removal of myelin debris (Cantoni *et al*, 2015). However, in mouse models for AD pathology, TREM2 deficiency resulted in inconsistent findings. Jay *et al* (2015) reported a detrimental role of TREM2 in AD by demonstrating that its knockout leads to a reduction in the amyloid plaque load, inflammation, astrogliosis, and tau phosphorylation. On the other hand, Wang *et al* (2015) demonstrated that TREM2 deficiency enhanced amyloid plaque load. This discrepancy may be due to the use of different mouse models, but also due to the fact that microglial function may be differentially compromised depending on the time point one investigates amyloid pathology (Tanzi, 2015).

It is well known that antibodies bound to amyloid plaques trigger Fc receptor‐mediated Aβ clearance by microglia cells (Bard *et al*, 2000). We were interested whether antibody‐mediated Aβ clearance may be influenced by TREM2 function. To avoid confounding effects of different mouse models, time points of analysis and differences in progression of disease pathology, which in aggressive APP/presenilin overexpressing mouse models may even override microglial amyloid plaque clearance, we choose several independent models to quantitatively investigate TREM2‐dependent antibody‐mediated Aβ clearance under controlled conditions. We used CRISPR/Cas9‐modified N9 microglial cell lines as well as bone marrow‐derived macrophages (BMDM) and primary microglia cells from wild‐type (wt) or *Trem2* knockout (ko) mice and investigated the potential of these cells for antibody‐dependent phagocytosis of pre‐formed Aβ fibrils or engulfment of antibody covered amyloid plaques from brain cryosections obtained from a mouse model for AD pathology.

## Results

### TREM2 deficiency reduces uptake efficacy of antibody‐bound Aβ by phagocytic cells

**Figure 1:**
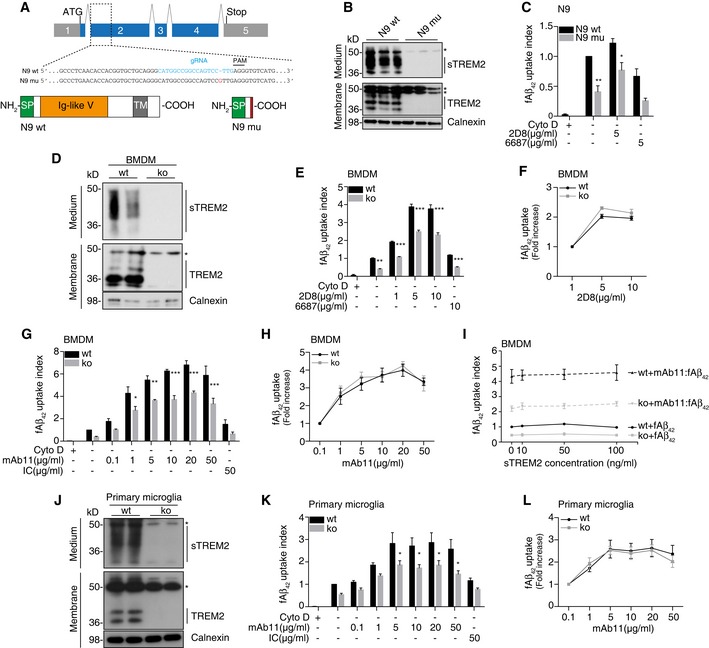
TREM2 deficiency reduces efficacy of antibody‐stimulated Aβ uptake by phagocytic cells Schematic of the mouse *Trem2* locus and the TREM2 protein. Sequence alignment of wild‐type N9 (N9 wt) and TREM2 mutant N9 (N9 mu) surrounding the gRNA target site. The gRNA sequence is in cyan, and protospacer‐adjacent motif (PAM) is marked with a line. The single nucleotide insertion is labeled in red. Schematic representation of wild‐type TREM2 (NP_112544.1) and CRISPR/Cas9‐modified TREM2 (N9 mu). TM, transmembrane domain; SP, signal peptide.Western blot analysis of lysates and media from wt and mutant N9 cells (N9 wt /mu) using the antibody anti‐murine TREM2 (clone 5F4), which is raised against the murine TREM2 extracellular domain. sTREM2, soluble TREM2. *indicate unspecific bands. Calnexin was used as a loading control.Phagocytosis of 1 μM HiLyte^™^ Fluor 488 Aβ_1‐42_ (fAβ_42_) by N9 wt and N9 mu in the presence or absence of antibody 2D8 or the non‐binding antibody 6687. Cytochalasin D (CytoD, 10 mM) was used as control to verify phagocytic uptake. (*n* = 4, ± SEM; two‐way ANOVA, interaction *P* = 0.61, genotype *P* < 0.0001, treatment *P* = 0.0001; *post hoc* tests wt vs. mu for the following conditions: fAβ_42_ *P* = 0.0043, fAβ_42_‐2D8 *P* = 0.0436).Western blot of BMDM derived from wt and *Trem2* knockout (ko) animals using antibody 5F4. *indicate unspecific bands.Phagocytosis of fAβ_42_ by BMDM from wt and *Trem2* ko animals in the presence or absence of 2D8, or the non‐binding control antibody 6687. (*n* = 3, ± SEM; two‐way ANOVA, interaction *P* = 0.0005, genotype *P* < 0.0001, treatment *P* < 0.0001; *post hoc* tests wt vs. ko for the following conditions: fAβ_42_ *P* = 0.0021, fAβ_42_‐2D8 1 μg/ml *P* < 0.0001, fAβ_42_‐2D8 5 μg/ml *P* < 0.0001, fAβ_42_‐2D8 10 μg/ml *P* < 0.0001, fAβ_42_/6687 10 μg/ml *P* = 0.0007).Quantification of relative fAβ_42_ uptake to lowest antibody concentration used (*n* = 3, ± SEM).Phagocytosis of fAβ_42_ by BMDM from wt and *Trem2* ko animals in the presence or absence of mAb11, or an isotype control antibody (IC). (*n* = 4, ± SEM; two‐way ANOVA, interaction *P* = 0.0223, genotype *P* < 0.0001, treatment *P* < 0.0001; *post hoc* tests wt vs. ko for the following conditions: fAβ_42_‐mAb11 1 μg/ml *P* = 0.0391, fAβ_42_‐mAb11 5 μg/ml *P* = 0.0069, fAβ_42_‐mAb11 10 μg/ml *P* < 0.0001, fAβ_42_‐mAb11 20 μg/ml *P* = 0.0001, fAβ_42_‐mAb11 50 μg/ml *P* < 0.0001).Quantification of relative fAβ_42_ uptake to lowest antibody concentration used (*n* = 4, ± SEM).Recombinant mouse sTREM2 does not rescue fAβ_42_ uptake in *Trem2*‐deficient BMDM. Increasing amounts of sTREM2 were added to the media of wt or *Trem2* ko BMDM in the presence or absence of mAb11 (10 μg/ml) (*n* = 4, ± SEM).Western blot of primary microglia from wt or *Trem2* ko animals using antibody 5F4. *indicate unspecific bands.Phagocytosis of fAβ_42_ by primary microglia from wt and *Trem2* ko animals in the presence or absence of mAb11, or an isotype control antibody (IC). (*n* = 5, ± SEM; two‐way ANOVA, interaction *P* = 0.4797, genotype *P* < 0.0001, treatment *P* < 0.0001; *post hoc* tests wt vs. ko for the following conditions: fAβ_42_‐mAb11 5 μg/ml *P* = 0.0449, fAβ_42_‐mAb11 10 μg/ml *P* = 0.0370, fAβ_42_‐mAb11 20 μg/ml *P* = 0.0299, fAβ_42_‐mAb11 50 μg/ml *P* = 0.0120).Quantification of relative fAβ_42_ uptake to lowest antibody concentration used (*n* = 5, ± SEM).Data information: (C, E, G, K) Quantification of internalized fAβ_42_ was normalized to wt without antibody. Bonferroni‐corrected pair‐wise *post hoc* tests were used.Source data are available online for this figure.

To investigate a potential influence of TREM2 deficiency on antibody‐mediated Aβ clearance, we first studied Aβ uptake in the microglial cell line N9 (Sessa *et al*, 2004). *Trem2* mutant cell lines were generated using the CRISPR/Cas9 technology (Ran *et al*, 2013). We obtained a mutant cell line with a single nucleotide insertion (93_94insG) within the target site, which leads to a frame shift and an early stop codon that eliminates TREM2 expression (Fig 1A). Immunoblotting confirmed the loss of full‐length, membrane‐bound TREM2 as well as soluble TREM2 (sTREM2) in *Trem2* mutant N9 cells (N9 mu) (Fig 1B). In line with our previous findings (Kleinberger *et al*, 2014), loss of TREM2 significantly reduced uptake of pre‐aggregated HiLyte^™^ Fluor 488‐labeled Aβ_42_ (fAβ_42_) (Fig 1C). Cytochalasin D, an inhibitor of actin polymerization, was used as negative control. Addition of 5 μg/ml of antibody 2D8, an antibody raised to the N‐terminus of the Aβ domain (Shirotani *et al*, 2007), significantly stimulated Aβ uptake in both N9 wt and N9 mu; however, phagocytosis remained less efficient in N9 mu (Fig 1C). As a negative control, we used antibody 6687 raised to the C‐terminus of APP (Capell *et al*, 2000). Although this antibody interacts with APP, it fails to increase Aβ uptake demonstrating that the specific interaction of 2D8 with Aβ is required to trigger the uptake (Fig 1C).

To investigate antibody‐stimulated Aβ clearance in primary phagocytic cells, we first used BMDM derived from *Trem2* ko animals (Turnbull *et al*, 2006) or wt controls. Western blotting confirmed the absence of sTREM2 and full‐length, membrane‐bound TREM2 in cells derived from *Trem2* ko mice (Fig 1D). We then studied fAβ_42_ uptake in the presence of 0, 1, 5, or 10 μg/ml of antibody 2D8 or a non‐binding control antibody 6687. BMDM readily internalized fAβ_42_, which could be blocked entirely by addition of cytochalasin D (Fig 1E). Consistent with our previous findings (Kleinberger *et al*, 2014), BMDM derived from *Trem2* ko mice showed a significantly reduced phagocytic activity compared to BMDM derived from wt mice (Fig 1E). Phagocytosis of fAβ_42_ in wt BMDM could be intensively stimulated by antibody 2D8 with a maximum stimulation at 5 μg/ml. Antibody 6687 even at a high concentration of 10 μg/ml had only a very minor effect (Fig 1E). Although antibody 2D8 significantly stimulated uptake of fAβ_42_ even in *Trem2* ko BMDM, phagocytosis was less efficient compared to wt at all antibody concentrations used (Fig 1E). These findings suggest that fAβ_42_ uptake is greatly stimulated upon antibody binding in both wt and *Trem2* ko BMDM; however, the overall uptake capacity is reduced in *Trem2* ko BMDM. In line with that, there was no significant change in the relative increase of antibody‐stimulated uptake in both genotypes (Fig 1F) suggesting that antibody‐stimulated uptake *per se* is not reduced due to TREM2 deficiency.

Next, we used the monoclonal antibody mAb11, a murine IgG2a antibody, which has similar amyloid binding properties like the therapeutically used human IgG1 anti‐Aβ antibody Gantenerumab (Bohrmann *et al*, 2012; Lathuiliere *et al*, 2016) and performed titration experiments in the fAβ_42_ uptake assay using BMDM derived from wt or *Trem2* ko mice. mAb11 but not an IgG2a isotype control (50 μg/ml) strongly stimulated phagocytosis of fAβ_42_ (Fig 1G). Interestingly, even very low concentrations of 0.1 μg/ml, which may be reached in the brain by peripheral antibody administration, were sufficient to trigger fAβ_42_ uptake. Uptake plateaued at 20 μg/ml and could not be further enhanced by using antibody concentrations up to 50 μg/ml (Fig 1G). In line with the data shown in Fig 1C and E, fAβ_42_ uptake by BMDM derived from *Trem2* ko mice could be efficiently stimulated by increasing the amounts of mAb11 (Fig 1G and H). However, again the phagocytic capacity never reached the level of wt BMDM even at the highest antibody concentration used (Fig 1G). Thus, a monoclonal antibody with efficient target engagement similar to the therapeutically used Gantenerumab stimulates both TREM2‐dependent and TREM2‐independent engulfment of fAβ_42_.

To investigate whether sTREM2 could rescue reduced fAβ_42_ uptake of BMDM derived from *Trem2* ko mice in a non‐cell autonomous manner, we supplemented the culture media of wt and ko cells with increasing amounts of recombinant mouse sTREM2. sTREM2 even added at a 10‐fold higher concentration as compared to its physiologically concentration in plasma of mice (approximately 10 ng/ml) did not rescue fAβ_42_ uptake efficacy of *Trem2* ko cells in the presence or absence of mAb11 (Fig 1I). Thus, receptor‐mediated signaling in a cell autonomous manner appears to trigger fAβ_42_ uptake.

Finally, we further confirmed our findings in primary microglia derived from wt or *Trem2* ko mice. Western blotting confirmed the absence of sTREM2 and full‐length, membrane‐bound TREM2 in primary microglia from *Trem2* ko mice (Fig 1J). In line with our previous findings (Kleinberger *et al*, 2014), microglia from *Trem2* ko mice showed significantly less uptake of fAβ_42_ (Fig 1K). Again, phagocytosis was similarly stimulated by mAb11 in a concentration‐dependent manner in both microglia derived from wt and microglia derived from *Trem2* ko mice (Fig 1K and L). In line with the above data derived from N9 cells and BMDM (Fig 1C, E, and G), total uptake capacity was reduced at each antibody concentration in *Trem2* ko microglia (Fig 1K).

### Increased Fcγ‐receptors expression and enhanced Syk phosphorylation in TREM2‐deficient BMDM

**Figure 2:**
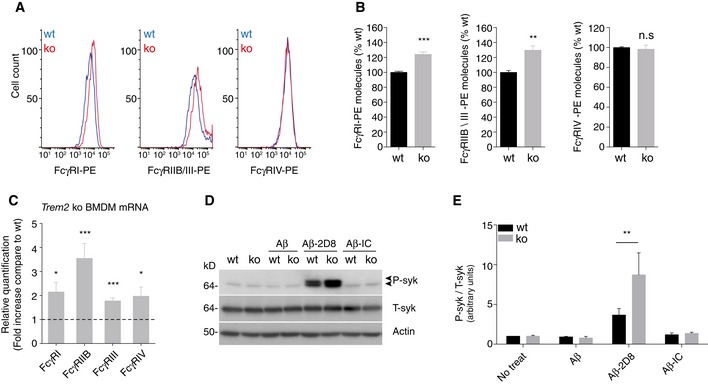
Increased Fcγ‐receptors expression and enhanced Syk phosphorylation in TREM2‐deficient BMDM Representative histograms for Fcγ‐receptors‐PE (FcγR‐PE) expression levels as used for quantification. Stacked histograms for log PE fluorescence intensity of wt and *Trem2* ko BMDM are shown for the respective FcγR (I, IIB/III, and IV).Relative quantification of cell surface levels of FcγR molecules. Absolute number of cell surface FcγR‐PE molecules was determined by the BD QuantiBRITE® method (see methods section for details) and normalized to expression levels of the respective wt control. (*n* = 4, ± SEM, *t*‐test, two‐tailed; wt vs. ko: FcγRI‐PE *P* = 0.0008, FcγRIIB/III‐PE *P* = 0.0033, FcγRIV‐PE *P* = 0.7001).mRNA levels of FcγR are increased in *Trem2* ko BMDM. Fold changes of the respective FcγR (I, IIB, III, IV) mRNA levels in *Trem2* ko BMDM were determined by quantitative real‐time PCR. (*n* = 6, ± SEM; one‐sample *t*‐test, two‐tailed; wt vs. ko: FcγRI *P* = 0.0118, FcγRIIB *P* = 0.0006, FcγRIII *P* = 0.0004, FcγRIV *P* = 0.0284)Phosphorylated Syk (P‐Syk) and total Syk (T‐Syk) levels were determined by Western blotting in lysates from wt and *Trem2* ko BMDM after 1 h treatment with Aβ alone, together with antibody 2D8 (Aβ‐2D8), or an isotype control (Aβ‐IC). Actin was used as a loading control.Quantification of P‐Syk normalized to T‐Syk. (*n* = 4, ± SEM; 2 way ANOVA, interaction *P* = 0.0490, genotype *P* = 0.0898, treatment *P* < 0.0001. Bonferroni‐corrected pair‐wise *post hoc* tests, **P = 0.0075 vs. wt). Source data are available online for this figure.

To investigate whether reduced phagocytic capacity but similar increases in antibody‐stimulated uptake in the absence of TREM2 may be due to compensatory mechanisms, we first investigated expression of Fcγ‐receptors (FcγR), which are of central importance for antibody‐mediated phagocytosis. Compared to wt BMDM, cell surface FcγRI, FcγRIIB, and (or) FcγRIII were significantly increased in *Trem2* ko cells (Fig 2A and B). Correspondingly, mRNA levels of the respective FcγR (I, IIB, III) were all significantly increased in *Trem2* ko cells (Fig 2C). mRNA level of FcγRIV was also increased, but it did not result in enhanced cell surface protein level (Fig 2A–C). To further validate the compensatory mechanisms, downstream signaling was studied. It is well known that upon stimulation of TREM2 as well as Fcγ‐receptors, Syk gets phosphorylated and activates a variety of downstream signaling cascades (Crowley *et al*, 1997; Paradowska‐Gorycka & Jurkowska, 2013; Ulrich & Holtzman, 2016). As expected, Syk phosphorylation strongly increases upon incubation of wt BMDM with 2D8‐bound Aβ, which was not the case when using an isotype control antibody together with Aβ (Fig 2D and E). Interestingly, phosphorylation of Syk is further increased upon stimulation with antibody‐bound Aβ in the absence of TREM2 (Fig 2D and E).

Together with increased levels of FcγR, this may suggest a compensatory increase in TREM2‐independent antibody/antigen uptake pathways probably explaining why *Trem2* ko cells still respond to increasing concentrations of anti‐Aβ antibodies.

### TREM2 deficiency reduces antibody‐mediated amyloid plaque clearance

**Figure 3:**
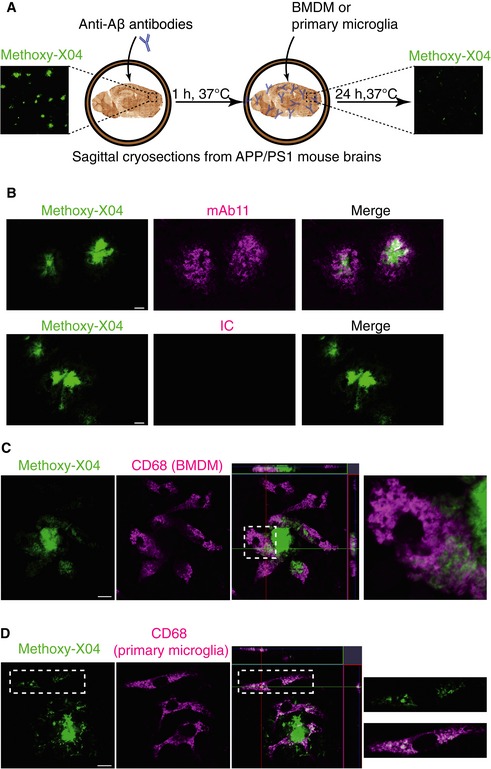
Engulfment of fibrillar Aβ by BMDM and primary microglia AA schematic shows 10‐μm cryosections of unfixed brain from 6‐month‐old APP/PS1 mice, which show a high amyloid plaque burden (right panel with methoxy‐X04 staining), were incubated with antibody for 1 h, followed by adding BMDM or primary microglia on top of the sections. After 24 h incubation, sections were analyzed by immunostaining or immunoblotting.BmAb11 but not the isotype control (IC) co‐localized with methoxy‐X04. Scale bar: 10 μm.C, DBMDM (C) or primary microglia (D) were cultured on cryosections pre‐incubated with mAb11 (1 μg/ml). After 24 h, sections were processed for immunostaining using antibody against CD68 to identify myeloid cells and methoxy‐X04 staining to visualize Aβ. Note that both cell types internalize Aβ into intracellular vesicles (right panels show enlargement of insets). Scale bar: 10 μm.

Next, we used an *ex vivo* model (Fig 3A), which would allow monitoring antibody‐dependent amyloid plaque clearance under controlled and comparable conditions (Bard *et al*, 2000). We used cryosections of whole brains from APP/PS1 mice (Radde *et al*, 2006) at 6 months of age, a time point where these mice are known to exhibit a high amyloid plaque burden within the brain (Radde *et al*, 2006). mAb11, but not the isotype control, binds selectively to the methoxy‐X04‐positive amyloid plaques (Fig 3B). When wt BMDM (Fig 3C) or primary microglia (Fig 3D) were added to brain sections pre‐incubated with mAb11, we observed a clustering of CD68‐positive cells around methoxy‐X04‐labeled amyloid plaques. Moreover, cells clustering around amyloid plaques engulfed Aβ fibers, as shown by the intracellular methoxy‐X04 staining, which partially co‐localized with the lysosomal protein CD68 (Fig 3C and D).

**Figure 4:**
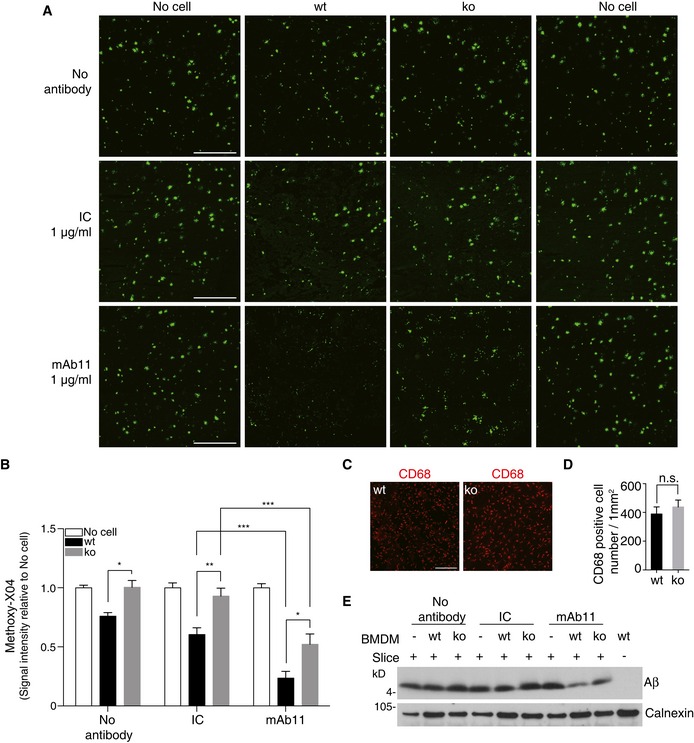
TREM2 deficiency reduces antibody‐mediated amyloid plaque clearance ABMDM from wt or *Trem2* ko mice were cultured on APP/PS1 mice brain cryosections incubated with or without mAb11 (1 μg/ml) or an isotype control (IC; 1 μg/ml) for 24 h. Sections were then probed with methoxy‐X04. Scale bar, 500 μm.BThe amyloid plaque load was quantified from the entire sagittal section. Sections incubated with medium (no cell) were set as baseline. (*n* = 6, ± SEM; two‐way ANOVA, interaction *P* < 0.0001, genotype *P* < 0.0001, treatment *P* < 0.0001; Tukey's multiple comparisons tests; wt vs. ko for the following conditions: no antibody *P* = 0.0304, IC *P* = 0.0049, mAb11 *P* = 0.0212; wt: IC vs. wt: mAb11 *P* = 0.0008; ko: IC vs. ko: mAb11 *P* = 0.0001).C, DEqual numbers of wt and *Trem2* ko BMDM were added, and cell numbers were analyzed after termination of experiments by quantifying the CD68‐positive cells on top of the sections. (*n* = 4, ± SEM; *t*‐test; n.s., non‐significant, *P* = 0.5004). Scale bar, 200 μm.EAβ was extracted by urea buffer from replicate slices of the experiment shown in (A), and total Aβ was identified by Western blotting. Source data are available online for this figure.

We next used the *ex vivo* assay to investigate TREM2‐dependent antibody‐stimulated amyloid plaque clearance. When cryosections of APP/PS1 mice were incubated with BMDM derived from wt or *Trem2* ko mice, a significant TREM2‐dependent reduction in amyloid plaque clearance was observed (Fig 4A and B). Upon pre‐incubation of cryosections with mAb11 (1 μg/ml), amyloid plaque clearance by wt BMDM was significantly stimulated (Fig 4A and B). In line with the results in Fig 1, TREM2‐deficient BMDM were significantly less efficient in clearing amyloid plaques than wt BMDM under the same experimental conditions (Fig 4A and B). Cell densities in the different experiments were similar, as assessed by CD68 staining on consecutive slices (Fig 4C and D). Reduced amyloid plaque clearance by BMDM derived from the *Trem2* ko animals upon antibody stimulation was confirmed by Western blotting of total protein lysates generated from cryosections after termination of the experiment. In line with the experiments in Fig 4A and B, mAb11 caused a stronger reduction in Aβ signals on Western blots in the presence than in the absence of TREM2. However, even in the absence of TREM2, a reduction in Aβ was observed with mAb11 pre‐incubation as compared to the no‐antibody control (Fig 4E).

### Improvement of amyloid plaque clearance by elevated antibody concentrations

**Figure 5:**
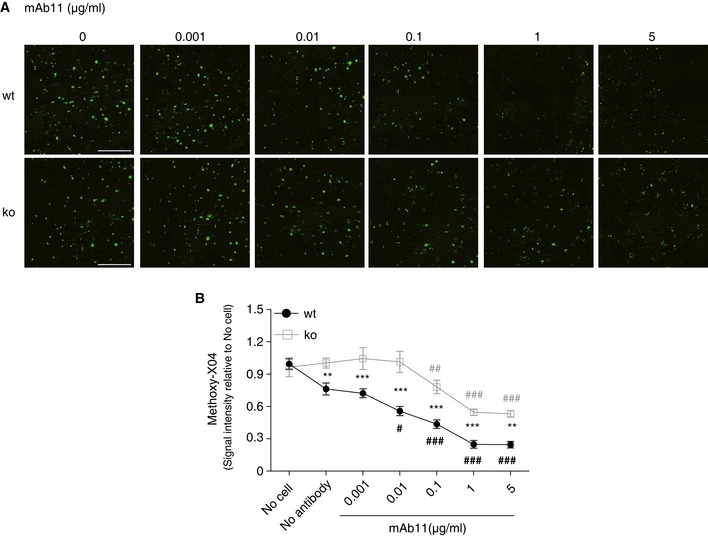
Compensation of reduced amyloid plaque clearance by elevated antibody dose Cryosections from unfixed brain of 6‐month‐old APP/PS1 mice were pre‐incubated with increasing concentrations of mAb11 (0.001, 0.01, 0.1, 1, 5 μg/ml). BMDM from wt or *Trem2* ko mice were added for 24 h. Sections were stained with methoxy‐X04. Scale bar, 500 μm.Methoxy‐X04 signals were quantified from the entire sagittal section. (*n* = 5, ± SEM; two‐way ANOVA, interaction *P* = 0.0082, genotype *P* < 0.0001, treatment *P* < 0.0001. Fisher's LSD *post hoc* comparisons; * show statistics between wt and ko under the same experimental condition. ^**#**^ in black shows wt compares to no‐antibody stimulation; ^#^ in gray shows ko compares to no‐antibody stimulation; wt vs. ko for the following conditions: no antibody *P* = 0.0053, mAb11 0.001 μg/ml *P* = 0.0003, mAb11 0.01 μg/ml *P* < 0.0001, mAb11 0.1 μg/ml *P* = 0.0001, mAb11 1 μg/ml *P* = 0.0007, mAb11 5 μg/ml *P* = 0.0011; following conditions compare to wt/no antibody: wt/mAb11 0.01 μg/ml *P* = 0.0166, wt/mAb11 0.1 μg/ml *P* = 0.0002, wt/mAb11 1 μg/ml *P* < 0.0001, wt/mAb11 5 μg/ml *P* < 0.0001; following conditions compare to ko/no antibody: ko/mAb11 0.1 μg/ml *P* = 0.0099, ko/mAb11 1 μg/ml *P* < 0.0001, ko/mAb11 5 μg/ml *P* < 0.0001.

We next titrated mAb11 with the aim to compare the efficacy of antibody‐mediated TREM2‐dependent amyloid plaque clearance. Cryosections from brains of APP/PS1 mice were pre‐incubated with increasing concentrations of mAb11 from 0.001 to 5 μg/ml before incubation with BMDM derived from wt or *Trem2* ko mice. In line with the fAβ_42_ uptake assays described in Fig 1, this revealed a concentration‐dependent clearance of amyloid plaques (Fig 5A and B). Comparison of the extend of methoxy‐X04‐positive amyloid labeling after clearance by BMDM derived from wt or *Trem2* ko mice demonstrates that antibody‐mediated clearance can occur in the absence of TREM2 (Fig 5A and B), similar to the uptake of fAβ_42_ shown in Fig 1. However, the total capacity to engulf amyloid plaques is reduced in *Trem2* ko BMDM (Fig 5A and B). Of note, a statistically significant effect on amyloid plaque clearance is observed in *Trem2* ko BMDM at 0.1 μg/ml, a concentration which is therapeutically reachable in brain by appropriate dose adjustment (Bohrmann *et al*, 2012; Lathuiliere *et al*, 2016). Taken together, our finding suggests that patients with compromised TREM2 function may require a higher dose of the therapeutic antibody to achieve efficient Aβ clearance.

## Discussion

A pivotal role of microglia and inflammatory mechanisms in AD and other neurodegenerative disorders, which was already emphasized early during AD research (McGeer & Rogers, 1992), was recently strongly supported by genetic evidence (Villegas‐Llerena *et al*, 2016). Specifically, rare heterozygous mutations in *TREM2*, which within the brain is exclusively expressed in microglia cells, dramatically increase the risk for late onset AD in a magnitude similar to *ApoE* ε*4* (Guerreiro *et al*, 2013; Jonsson *et al*, 2013). Although TREM2 is well known to be involved in phagocytosis and removal of apoptotic neurons (Takahashi *et al*, 2005; Colonna *et al*, 2007; Hsieh *et al*, 2009), the pathological consequences of a TREM2 loss of function in the context of AD pathogenesis are highly controversial. While Aβ was shown to be engulfed at least to some extend in a TREM2‐dependent manner in cultured cells (Kleinberger *et al*, 2014), experiments on mouse models for AD pathology reached surprising and opposite results. On the one hand, reduction in TREM2 ameliorated several aspects of AD pathology, including inflammation, astrocytosis, and amyloid plaque burden (Jay *et al*, 2015), while on the other hand, *Trem2* deficiency leads to exacerbated disease pathology including increased amyloid plaque burden (Wang *et al*, 2015). However, attenuated Aβ engulfment by microglia was thought to be due to reduced migration and survival and thus not directly associated with phagocytic activity of microglial cells (Wang *et al*, 2015). Our findings may now provide direct evidence that TREM2 is required at least partially for Aβ engulfment and clearance. Even in the absence of antibody stimulation, we consistently find reduced Aβ uptake and amyloid plaque clearance in all model systems used. Potential survival deficits in *Trem2* ko cells do not impact our results, since uptake was measured after 2 h of incubation in the phagocytosis assay. Moreover, equal amounts of cells were used in the *ex vivo* plaque clearance assay and cell density was assessed after termination of experiments ruling out differences due to survival or the total number of phagocytic cells.

After antibody binding to Aβ, uptake and amyloid plaque clearance increase in a concentration‐dependent manner in the presence or absence of functional TREM2, although the total uptake capacity of cells lacking TREM2 is reduced. This indicates that TREM2‐independent Fcγ‐receptor‐mediated pathways are intact and used in addition to TREM2‐dependent uptake mechanisms. Indeed, it has been shown previously that Syk phosphorylation is an important signaling event upon Fcγ‐receptors activation in macrophage phagocytosis (Greenberg *et al*, 1994; Crowley *et al*, 1997). Higher levels of FcγR and upregulated phosphorylated Syk in *Trem2* ko BMDM upon stimulation with Aβ‐2D8 immune complexes may therefore suggest a compensatory upregulation of TREM2*‐*independent phagocytosis.

Our findings might also indicate that direct effects of TREM2 on amyloid plaque clearance are difficult to assess *in vivo* since in the absence of an antibody stimulus Aβ uptake is 4–5 fold lower and probably difficult to quantify. In addition, the mouse models used to study the effects of TREM2 on amyloid plaque clearance not only highly overexpress APP but also express rather aggressive familial AD‐associated mutations, which may override modulatory effects of TREM2. Moreover, since TREM2 affects survival of microglial cells (Wang *et al*, 2015; Wu *et al*, 2015), the age as well as the specific mouse model used for the investigation must be carefully considered. In that regard, it is interesting to note that at very early time points of amyloid plaque deposition, Aβ accumulation was similar in wt and *Trem2* ko mice, but *Trem2* ko mice showed reduced microglia accumulations around amyloid plaques (Wang *et al*, 2016).

Finally, our findings may be valid for future immunotherapeutic approaches. Anti‐Aβ immunotherapy is currently a promising and clinically advanced approach (Wisniewski & Goni, 2015). It not only lowers the amyloid plaque load, but also prevents *de novo* deposition of plaques and stabilized memory deficits at least to some extend in two recently reported clinical trials (Reardon, 2015). We now demonstrate that mAb11, a murine IgG2a antibody, which has similar amyloid binding properties like Gantenerumab (Lathuiliere *et al*, 2016) which is currently explored in clinical trials (Bohrmann *et al*, 2012), significantly stimulates Aβ engulfment even in the absence of TREM2. For successful immunotherapy, knowledge on microglial activity and survival in individual patients may be crucial for optimal outcome of the treatment. In that regard, we recently demonstrated that in AD patients, sTREM2 levels significantly increase very early before onset of AD and tend to decrease in later phases of the disease (Suarez‐Calvet *et al*, 2016). In addition to CSF biomarkers, microglial PET imaging may be required similar to amyloid PET imaging used in the clinic to select patients for enrollment into clinical trials and to *in vivo* prove the effects of treatment on amyloid plaque load. If microglial function and survival are reduced during aging in a TREM2‐dependent manner, one may also speculate to modulate TREM2 activity. Moreover, in case of attenuated TREM2 function, an increased dose of therapeutic antibodies may be required to compensate impaired plaque clearance.

## Materials and Methods

### Mice

All animal experiments were performed in accordance with local animal handling laws. APP/PS1 (Radde *et al*, 2006) and *Trem2* knockout mice (*Trem2* ko) (Turnbull *et al*, 2006) were maintained on a C57BL/6J background. For bone marrow extraction, adult mice (aged from 2 to 12 months, mixed gender) were euthanized by CO_2_ and then sacrificed by cervical dislocation. For primary microglia culture, postnatal day P0‐2 mice (mixed gender) were sacrificed by decapitation.

### CRISPR/Cas9‐mediated genome engineering in N9

The murine microglia cell line N9 (Sessa *et al*, 2004) was maintained in Dulbecco's modified Eagle's medium (DMEM) + GlutaMAX^™^ (Life Technologies) with 10% (v/v) fetal calf serum (FCS; Sigma‐Aldrich) and 100 U/ml penicillin, 100 μg/ml streptomycin. All cells were mycoplasmas free. N9 cells were transfected with pSpCas9(BB)‐2A‐Puro V2.0 (PX459; gift from Feng Zhang; Addgene plasmid # 62988) using the Nucleofector^™^ SF‐Kit for 4D‐Nucleofector^™^ (Lonza) according to manufacturer's recommendations. Twenty‐four hours post‐transfection, cells were selected with 4 μg/ml puromycin for 2 days. Single cell clones were then cultured with normal culture medium, followed by screening for genetic modifications in *Trem2* by PCR amplification and missense detection using T7 endonuclease I (NEB). Mutations were confirmed by direct sequencing (GATC‐Biotech).

### Bone marrow‐derived macrophages culture

**Figure EV1:**
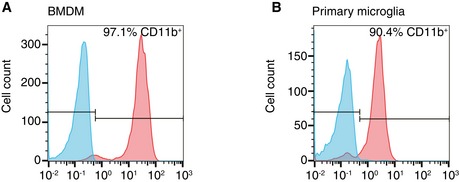
BMDM and primary microglia are positive for CD11b Surface staining of CD11b was analyzed using flow cytometry. 
About 97.1% bone marrow‐derived macrophages (BMDM) were positive for CD11b.Primary microglia from mixed glial culture were 90.4% positive for CD11b.

Bone marrow‐derived macrophages (BMDM) were prepared essentially as described before (Marim *et al*, 2010). Briefly, mice femurs and tibias were dissected and sterilized with 70% ethanol. The bones were flushed with a syringe filled with advanced RPMI 1640 (Life Technologies) to extrude bone marrow. Cell suspensions were filtered through a 100‐μm cell strainer and incubated for 2 min in ACK lysis solution (Thermo Fisher Scientific) to lyse red blood cells. The bone marrow cells were cultured and differentiated in advanced RPMI 1640 supplemented with 2 mM l‐glutamine, 10% (v/v) FCS, 100 U/ml penicillin, 100 μg/ml streptomycin, and 50 ng/ml murine M‐CSF (R&D System) using non‐tissue culture treated Petri dishes (BD Biosciences). Three days after seeding, fresh murine M‐CSF (final concentration of M‐CSF 50 ng/ml) was added and media was changed at day 5 of culture. BMDM were used for experiments at day 7 *in vitro*. Immunostaining with CD68 (Figs 3C and 4C) as well as flow cytometry (see below) using anti‐CD11b antibody (Fig EV1A) was used to confirm the identity of these cells.

### Primary microglial culture

Primary murine microglial cultures were prepared as previously described (Fleisher‐Berkovich *et al*, 2010). Briefly, mixed glial cultures were prepared from the whole brain devoid meninges and cerebellum of P0‐2 mice and cultured in DMEM with Glutamax I (Life Technologies), supplemented with 10% (v/v) FCS, 100 U/ml penicillin and 100 μg/ml streptomycin. About 50% of the medium was replaced every other day, and 10 ng/ml murine M‐CSF (R&D System) was supplemented at day 7 in culture. After 10 days in culture, cells were shaken using horizontal orbital shaker at 200 rpm for 30 min to isolate microglia. The medium containing microglia was pelleted at 200 g for 8 min and reseeded to corresponding plates for experiments. Cell identity was confirmed using anti‐CD68 (Fig 3D) and anti‐CD11b (Fig EV1B) antibodies.

### Flow cytometry

Bone marrow‐derived macrophages or primary microglial cells were suspended in FACS buffer (Hank's balanced salt solution, no calcium, no magnesium; 0.2% bovine serum albumin; Thermo Fisher Scientific). Samples were labeled with anti‐CD11b antibodies (M1/70), APC‐Cy7^®^ conjugated (1:100; Life Technologies) for 30 min at 4°C. Data were acquired using MACSQuant^®^ VYB (Miltenyi Biotec) and analyzed by FlowJo software.

To quantify absolute expression levels for the different cell surface Fcγ‐receptors (FcγR), BMDM were labeled with PE anti‐FcγRII/III (1:100; BD Bioscience), PE anti‐FcγRI (1:100; R&D System) or PE anti‐FcγRIV (1:100; BD Bioscience) at 4°C for 30 min. Data were acquired on a FACSVerse flow cytometer (BD, USA) and analyzed using FACSuite software (BD, USA). Absolute numbers of PE anti‐FcγR molecules per cell were determined using QuantiBRITE (BD Bioscience) PE calibration beads following the analysis strategy according to the manufacturer's protocol. Gates were set according to unlabeled and isotope controls. Absolute PE anti‐FcγR molecules counts were performed per individual sample and normalized to the mean of the wt control group.

### Cell lysis and immunoblotting

Western blot analysis of membrane‐bound TREM2 was performed as previously described (Kleinberger *et al*, 2014). For Western blot analysis of Aβ, lysates were prepared with 8 M urea lysis buffer (8 M Urea, 50 mM Tris–HCL pH 7.4) freshly supplemented with a protease inhibitor cocktail (Sigma‐Aldrich) and incubated on ice for 30 min. The lysate was mixed with Laemmli sample buffer supplemented with beta mercaptoethanol, and equal amounts of protein were separated by SDS–PAGE. After transfer onto polyvinylidene difluoride membranes (Amersham Hybond P 0.45 PVDF, GE Healthcare Life Science) or nitrocellulose membranes (GE Healthcare Life Science), blots were blocked for 1 h with I‐Block^™^ (Thermo Fisher Scientific) and exposed to 5F4 for TREM2 and sTREM2 or 2D8 (Shirotani *et al*, 2007) for Aβ. Signals were visualized with HRP‐conjugated secondary antibodies using ECL kit (Thermo Fisher Scientific).

### Antibodies

For *in vitro* and *ex vivo* phagocytosis assay, the following antibodies were used: rat monoclonal antibody 2D8 against Aβ_1–16_ (Shirotani *et al*, 2007), rabbit polyclonal antibody 6687 against cytosolic domain of APP (Capell *et al*, 2000), mouse IgG2a isotype control (Sigma‐Aldrich), and mouse monoclonal mAb11 (IgG2a (Bohrmann *et al*, 2012; Lathuiliere *et al*, 2016)). mAb11 is a closely related clone to the clinically used anti‐Aβ antibody Gantenerumab that binds preferentially to Aβ 40/42 aggregates, namely Aβ fibrils and oligomers via a conformational epitope comprising N‐terminal and central epitopes (more details on epitopes in (Bohrmann *et al*, 2012; Lathuiliere *et al*, 2016)). Affinity parameters were determined before for both antibodies by surface plasmon resonance (Biacore) and revealed equilibrium dissociation constant (KD) values for mAb11 between 0.14 and 0.67 nM, which were determined for Aβ40 and Aβ42, which is similar to Gantenerumab with a KD of 0.6 nM (Bohrmann *et al*, 2012; Lathuiliere *et al*, 2016). Binding and stability of bound antibody to soluble Aβ monomers is substantially lower. We measured a KD of 18 nM for Gantenerumab binding to Aβ monomers with a notably kinetically instable complex with monomers showing a rapid off‐rate. In more details, the kinetically most stable complex formation with Gantenerumab was observed for Aβ fibrils (kd 2.8 × 10^−4^ 1/s) and Aβ oligomers (kd 4.9 × 10^−4^ 1/s), whereas for monomeric Aβ, a higher dissociation rate was observed (kd 1.2 × 10^−2^ 1/s) suggesting rapid exchange of antibody‐bound monomeric Aβ. Thus, mAb11 binding parameters can be considered almost identical to Gantenerumab. Selective binding to aggregated Aβ40/42 is in agreement with demonstrated amyloid plaque binding measured by immunofluorescence *in vitro* and *in vivo*. Selective and sensitive binding to amyloid plaques in human AD and transgenic mouse brain tissues is again very comparable for Gantenerumab and mAb11 with positive immundecoration detectable at low concentration of 10 ng/mL and plaque binding *in vivo* in transgenic mice (Bohrmann *et al*, 2012; Lathuiliere *et al*, 2016).

For immunofluorescence staining of BMDM and primary microglia, staining was performed using rat anti‐CD68 (1:500; AbD Serotec) and goat anti‐rat Alexa Fluor 555 (1:200; Thermo Fisher Scientific).

For Western blotting of TREM2, we raised a rat monoclonal antibody (clone 5F4; 1:50) against the extracellular domain of murine TREM2 (Creative Biomart; Trem2‐3276M). Further used antibodies were rat antibody 2D8 (1:100) (Shirotani *et al*, 2007) against Aβ, rabbit anti‐calnexin (1:3,000; Enzo Life Sciences), secondary antibodies HRP‐conjugated goat anti‐rat (1:5,000; Santa Cruz Biotechnology), and goat anti‐rabbit IgG (1:10,000; Promega).

### *In vitro* Aβ phagocytosis assays

Phagocytosis assays with N9, BMDM, and primary microglia were performed as described (Kleinberger *et al*, 2014) with minor adjustments. Briefly, HiLyte^™^ Fluor 488 Aβ_1‐42_ (Anaspec) was aggregated overnight at 37°C with agitation. 3 × 10^4^ N9 cells, BMDM, or 2 × 10^4^ primary microglia were plated in poly‐D‐lysine‐coated black‐walled 96‐well plates (Greiner bio‐one). Pre‐aggregated HiLyte^™^ Fluor 488 Aβ_1‐42_ (fAβ_42_) was incubated with antibodies for 1 h at 37°C. Fibril Aβ_42_ or antibody‐fAβ_42_ complexes were added to a final concentration 1 μM and incubated for 2 h. As negative control, 10 mM cytochalasin D was added 30 min before addition of fAβ_42_ or antibody‐fAβ_42_. Before measurement, medium was removed and extracellular fAβ_42_ was quenched with 100 μl 0.2% trypan blue in phosphate‐buffered saline (PBS), pH 4.4 for 1 min. After aspiration, fluorescence signals were measured at 485‐nm excitation/538‐nm emission using a Fluoroskan Ascent^™^ Microplate Fluorometer (Lab Systems).

For the experiment shown in Fig 1I, BMDM were incubated with recombinant mouse sTREM2 (Creative Biomart) overnight before adding fAβ_42_ or antibody‐fAβ_42_ complexes.

### *Ex vivo* plaque clearance assay

*Ex vivo* plaque clearance assays were performed as previously described (Bard *et al*, 2000) with the following slight modifications. 6‐month‐old APP/PS1 transgenic mice (mixed gender) were perfused with PBS. Brains were separated into two hemispheres and snap‐frozen using dry ice powder. Frozen brains were sectioned into 10‐μm sagittal sections using a cryostat (Leica) and collect onto poly‐D‐lysine‐coated round glass coverslips (15 mm). Sections were dried at room temperature for 2 h followed by incubation with antibodies (isotype control (IC) or anti‐Aβ) in culture medium for 1 h at 37°C. BMDM or primary microglia cells were seeded at densities of 3×10^5^ cells per well in 12‐well plates and incubated at 37°C with 5% CO_2_ for 24 h. After incubation, cultures were fixed with 4% paraformaldehyde for 15 min and stained with methoxy‐X04 (2 μg/ml; Tocris Bioscience). In the indicated experiments, the fixed cultures were permeabilized with 0.1% Triton X‐100 for 3 min and stained with rat anti‐CD68 antibody overnight at 4°C followed by methoxy‐X04 staining.

Images were acquired on a LSM700 confocal microscope with Z stacks and tile scan to cover the entire sagittal sections using the Zen 2009 imaging software (Zeiss). Maximum intensity projection from each sagittal sections was then analyzed by ImageJ (Schneider *et al*, 2012) to obtain methoxy‐X04 signal intensities. In Fig 4A and B, four consecutive slices were set as a group, and signals from slices incubated with no cell were set as base line. Methoxy‐X04 signal intensities from slices either incubated with wt or incubated with *Trem2* ko BMDM were compared to base line. In Fig 5A and B, eight consecutive slices (#1–8) were used as a group and signal intensities from slices #1 and 8 were set as base line. Signals from slices #2 to 6, which were incubated with increasing concentrations of mAb11 (0–5 μg/ml) in the presence of BMDM, were compared to base line.

### Quantitative real‐time PCR analysis

Total RNA was isolated from BMDM using the RNeasy Mini Kit (Qiagen) according the manufacturer's instructions. The concentration and purity of RNA was determined using the NanoPhotomer (Implen). Equal amounts of RNA were reverse‐transcribed into cDNA using High‐capacity cDNA Reverse Transcription kit (Thermo Fisher). The expression level of Fcγ‐receptors (I, IIB, III, IV) was analyzed by Taqman^®^ real‐time PCR assay. Probes target to FcγΙ (Mm00438874_m1, Thermo Fisher Scientific), or FcγΙΙB (Mm00438875_m1, Thermo Fisher Scientific), or FcγΙΙΙ (Mm00438882_m1, Thermo Fisher Scientific), or FcγΙV (Mm00519988_m1, Thermo Fisher Scientific) were mixed with 1:5 diluted cDNA, added to the Taqman^®^ master mix (Thermo Fisher Scientific), and amplified with the 7500 fast real‐time PCR system (Applied Biosystems). Geometric mean of two housekeeping genes, Gusb (Mm01197698_m1, Thermo Fisher Scientific) and Hsp90ab1 (Mm00833431_g1, Thermo Fisher Scientific), which highly correlate with each other, was used as endogenous control. Fold change of mRNA in *Trem2* ko BMDM was calculated using wt BMDM as reference sample.

### Statistics

All statistical analysis was performed using Prism 6 (GraphPad). Data are presented as mean ± SEM with biological repeats. Data from the phagocytosis assays were analyzed by two‐way ANOVA with Bonferroni correction for multiple comparison. Cell surface FcγR relative levels were analyzed by Student's *t*‐test. Fold change of mRNA was analyzed by one‐sample *t*‐test of its log2 value. Western blot quantification in Fig 2C was analyzed by two‐way ANOVA with Bonferroni correction for multiple comparison. Methoxy‐X04 signal intensity presented in Figs 4B and 5B was analyzed by two‐way ANOVA, with Tukey's and Fisher's least significant difference (LSD) *post hoc* tests for pair‐wise comparisons, respectively. All tests were 2‐tailed, with a significant level of α = 0.05.

## Author contributions

XX, GK, and CH designed the study and interpreted the results. XX performed all experiments except generating mutant N9 cells, which was performed by GW and AC. The quantification of cell surface Fcγ‐receptors expression level was done by AL. BB, and IK provided antibodies. RF generated monoclonal antibody against TREM2 (clone 5F4). FM and GK provided bone marrow cells. CH wrote the manuscript with input of all co‐authors.

## Conflict of interest

C.H. is an advisor of F. Hoffmann‐La Roche. I.K. and B.B. are full‐time employees at Roche. All other authors declare that they have no conflict of interest.

**The paper explained**

**Problem**

Immunotherapeutic approaches are currently the most advanced treatments for Alzheimer's disease (AD). Antibodies against amyloid β‐peptide (Aβ) bind to amyloid plaques and trigger their clearance by microglia via Fc receptor‐mediated phagocytosis. Rare variants in the triggering receptor expressed on myeloid cells 2 (*TREM2*) increase the risk for late onset AD. Since a loss of *Trem2* function reduces the ability of microglia to engulf Aβ, we investigated whether antibody‐mediated Aβ clearance may be affected by TREM2 deficiency.

**Results**

Anti‐Aβ antibodies stimulate Aβ uptake and amyloid plaque clearance in a dose‐dependent manner in the presence or absence of TREM2. Elevated levels of Fc receptors in TREM2‐deficient cells indicate compensatory mechanisms, which stimulate the phagocytic activity in the absence of TREM2. Albeit compensatory increases of Fc receptor‐mediated phagocytosis, TREM2‐deficient phagocytic cells, showed significantly reduced uptake of antibody‐bound Aβ and as a consequence reduced clearance of amyloid plaques. Titration experiments revealed that reduced efficacy of amyloid plaque clearance by *Trem2* knockout cells can be improved by elevating the concentration of therapeutic antibodies.

**Impact**

Our findings are of direct therapeutic relevance, since they suggest that patients with phagocytic deficits caused by loss of TREM2 function may need a higher immunotherapeutic antibody dose to efficiently clear amyloid plaques. Thus, monitoring microglia function in patients at risk for AD may facilitate efficient immunotherapeutic strategies. In addition, our study unambiguously proofs that TREM2 function is required for Aβ uptake and amyloid plaque clearance.

## Supporting information



Expanded View Figures PDF

Review Process File

Source Data for Figure 1

Source Data for Figure 2D

Source Data for Figure 4E
