# Supplementary material for: TREM2 deficiency reduces the efficacy of immunotherapeutic amyloid clearance
Source: EMBO Mol Med. 2016 Jul 8;8(9):992–1004. doi: 10.15252/emmm.201606370 (PMC5009806; doi:10.15252/emmm.201606370)
Supplement: Supplementary file 1 — Expanded View Figures PDF [file EMMM-8-0992-s001.pdf]

Expanded View Figures

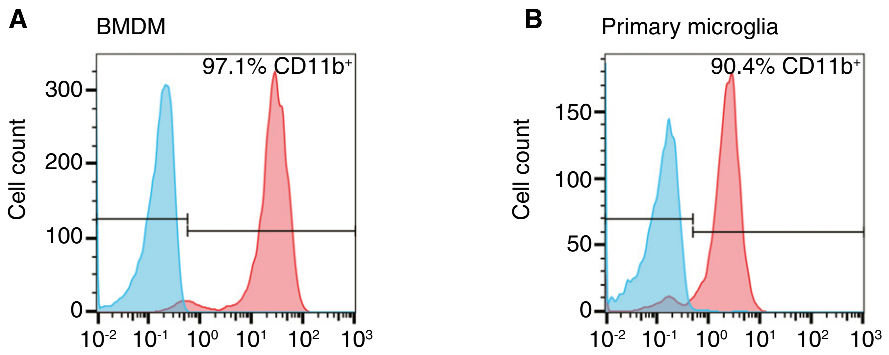

Figure EV1. BMDM and primary microglia are positive for CD11b.

Surface staining of CD11b was analyzed using flow cytometry.

A About 97.1% bone marrow-derived macrophages (BMDM) were positive for CD11b.

B Primary microglia from mixed glial culture were 90.4% positive for CD11b.

© 2016 The Authors
