# Supplementary figures and images for: TREM2 deficiency reduces the efficacy of immunotherapeutic amyloid clearance
Source: EMBO Mol Med. 2016 Jul 8;8(9):992–1004. doi: 10.15252/emmm.201606370 (PMC5009806; doi:10.15252/emmm.201606370)

**B.**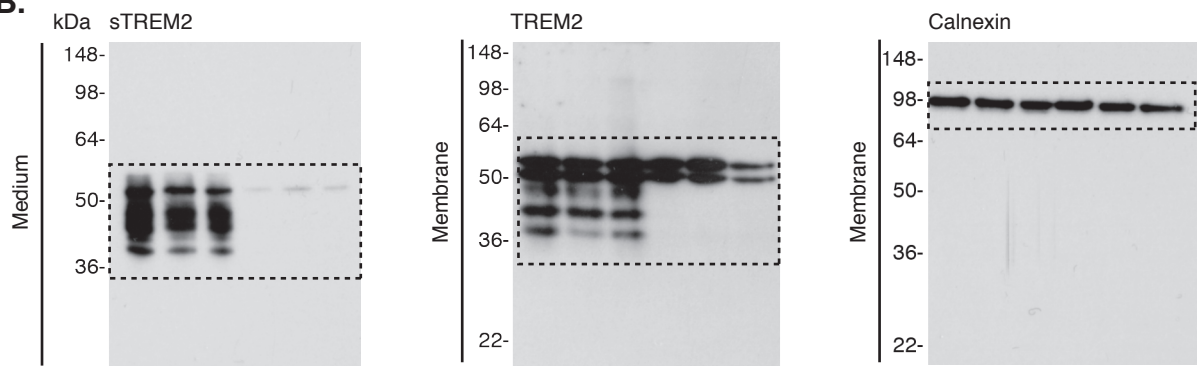**D.**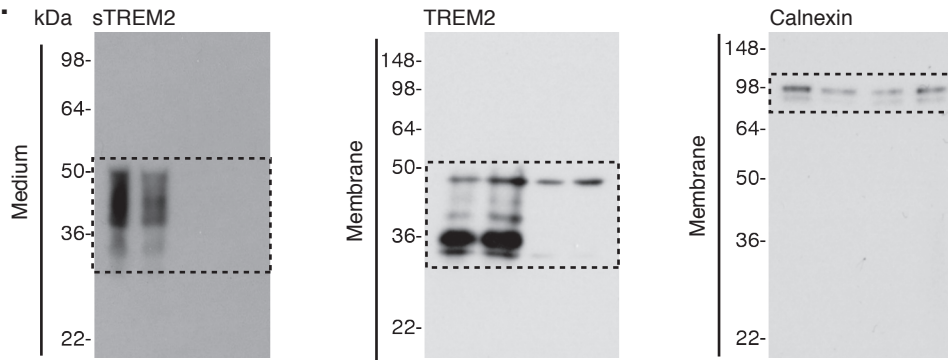**J.**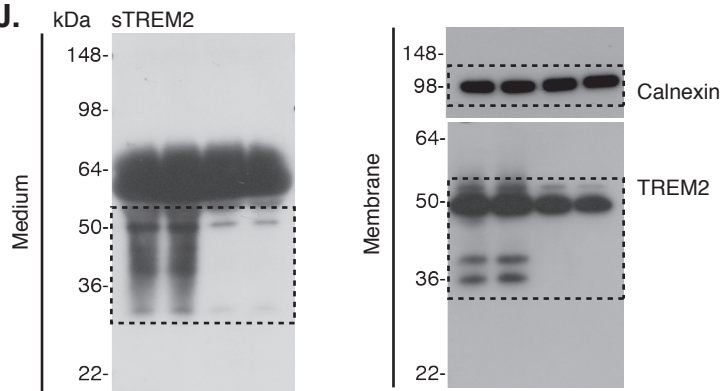

Xiang *et al.*, Fig. 1 Source data

Supplement: Supplementary file 3 — Source Data for Figure 1 [file EMMM-8-0992-s002.pdf]

D.

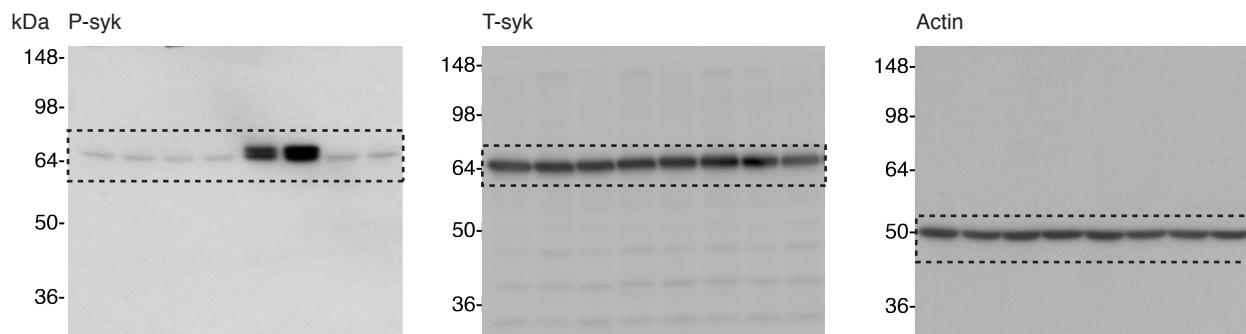

Xiang *et al.*, Fig. 2 Source data

Supplement: Supplementary file 4 — Source Data for Figure 2D [file EMMM-8-0992-s003.pdf]

**E.**

kDa A $\beta$

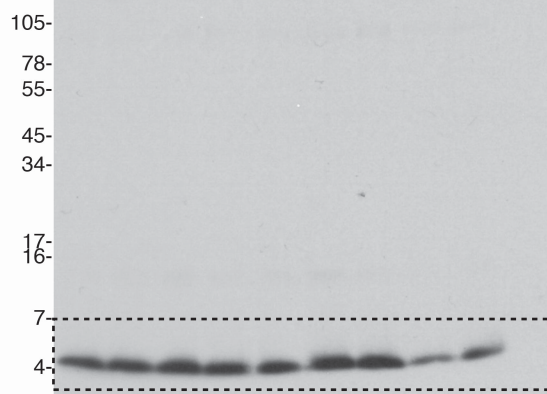

Calnexin

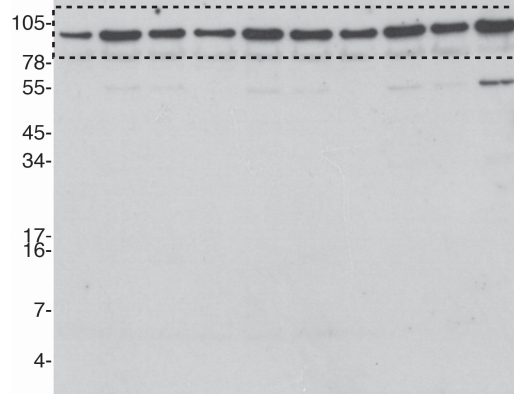

Xiang *et al.*, Fig. 4 Source data

Supplement: Supplementary file 5 — Source Data for Figure 4E [file EMMM-8-0992-s004.pdf]
