# Supplementary material for: TREM2 deficiency reduces the efficacy of immunotherapeutic amyloid clearance
Source: EMBO Mol Med. 2016 Jul 8;8(9):992–1004. doi: 10.15252/emmm.201606370 (PMC5009806; doi:10.15252/emmm.201606370)
Supplement: Supplementary file 2 — Review Process File [file EMMM-8-0992-s005.pdf]

TREM2 deficiency reduces the efficacy of immunotherapeutic amyloid clearance

Xianyuan Xiang, Georg Werner, Bernd Bohrmann, Arthur Liesz, Fargol Mazaheri, Anja Capell, Regina Feederle, Irene Knuesel, Gernot Kleinberger and Christian Haass

Corresponding author: Christian Haass, Ludwig-Maximilians-University DZNE

Review timeline:

Submission date:	10 March 2016
Editorial Decision:	11 April 2016
Revision received:	19 May 2016
Editorial Decision:	08 June 2016
Revision received:	09 June 2016
Accepted:	10 June 2016

Transaction Report:

(Note: With the exception of the correction of typographical or spelling errors that could be a source of ambiguity, letters and reports are not edited. The original formatting of letters and referee reports may not be reflected in this compilation.)

Editor: Céline Carret

1st Editorial Decision 11 April 2016

Thank you for the submission of your manuscript to EMBO Molecular Medicine. We have now heard back from the three referees whom we asked to evaluate your manuscript.

You will see that referees 1 and 3 are fully supportive of the study. However, referee 2 raises a certain number of technical issues that are, we believe, addressable within our 3-months deadline for major revisions. We would like to strongly encourage you to address all comments and resubmit your revised article for further consideration. As you know, it is EMBO Molecular Medicine policy to allow only a single round of revision and that, as acceptance or rejection of the manuscript may depend on another round of review, your responses should be as complete as possible.

I look forward to receiving your revised manuscript.

***** Reviewer's comments *****

Referee #1 (Remarks):

This is an interesting manuscript addressing the important question of how exactly TREM2 mutations increase the risk of developing AD. A set of in vitro and ex vivo experiments convincingly demonstrates that loss of TREM2 reduces antibody dependent clearance of Abeta fibrils. Overall, these data support the notion that loss of TREM2 function may cause AD through decreased amyloid clearance. This reviewer would like to suggest three points for further clarification:

(1) Did the authors look in their in vitro/ex vivo assays for the role of soluble TREM2, i.e. supplementation of knockout cells with soluble TREM2 from wt cells etc.? This would be very informative for understanding the role - if any - of this molecule in the overall scheme.
(2) Non-specialists would benefit from an even clearer discussion of the discrepancy with the TREM2 knockout mouse study reporting reduced pathology. Would the authors suggest that cross breeding a TREM2 KO mouse with a relatively mild Ab overproducer (e.g. TG2576)should lead to increased amyloid pathology at relatively early age (e.g. around 12 months)?
(3)I would rephrase the last sentence of the results suggesting that the data of the ex vivo assay indicate that TREM2 patients would benefit from amyloid immunotherapy, because the required antibody concentration of the ex vivo assay would be reachable - the assay is after all contrived and does not reflect in vivo human microglia. I think it is fair to state that such patients would likely require higher antibody doses, but whether these doses are reachable in the clinic is hard to know and not critical for the main conclusion of the present study.

Referee #2 (Remarks):

While parts of the manuscript are interesting, but at times, is not written very scientifically/specifically when 'similar properties', 'stronger reduction of Abeta signals' and other vague terms are used. There are several major points that raise serious concerns.

Major comments:
1. The mAb11 antibody was used, which has not been described in the manuscript. An 'irrelevant antibody 6687 raised to the C-terminus of APP was used as a negative control'. What does irrelevant mean here? It would be more appropriate to include other antibodies, which recognize specific Abeta epitopes. Also, A11, according to a statement given in the manuscript, has 'similar properties' to Gantenerumab, which is supposed to recognize fibrils as described in ref 36. Depending on what isoforms of Abeta and aggregate states A11 is recognizing it could explain one or the other result presented without TREM2 being involved, especially Western blot data shown in figure 4. There is no reference given where A11 has been characterized in detail, or one that may not rely on oligomer-fibril recognition.

2. This manuscript is using quite artificial systems, based on the use of a fluorescently labelled Abeta42 that we are expected to supposedly exist as aggregates, then coincubated with various antibodies, which is then applied to purified microglia or BMDM cultures, or glia co-cultured with brain slices. Does this fluorescent tag modify the aggregation behaviour or binding of the antibodies? Are similar results obtained using non-fluorescently labelled Abeta42? Is Abeta42 the only species of amyloid to play a role in toxicity/pathology of Alzheimer's disease?

3. Microglia and BMDM were cultured with brain slices from APP/PS1 mice, however, no marker is used at any point to identify these cells like cell-specific markers, other than CD68. What regions of the brain were used to prepare slices? Furthermore, whether any resident microglia or BMDM present in the brain slices used, were not assessed prior to the co-culturing with exogenously added glia. Is there a contribution of these resident glial cells to the phagocytosis of Abeta-antibody complexes? Since TREM2 has been shown to affect microglia survival and these amyloid uptake assays on slices lasted 24 hours (post cell seeding), how can the authors ascertain that the seeded microglia are responsible for amyloid uptake? What is the survival of TREM2-/- microglia and BMDM?

5. Syk is postulated to be a downstream effector of TREM2. In figure 2, the levels of phosphorylated Syk were normalized only with actin. Generally, one should determine the proportion of total Syk that was phosphorylated. Furthermore, this seems like a piece of hanging data. The role or contribution of Syk is not further explored in this antibody-mediated uptake of Abeta42.

6. There are relevant details regarding experimental design that are missing. For example, in Figure 4, what marker was used to determine cell number? This is not found in the figure legend nor in the figure itself.

Other comments:
1. The title of the manuscript is misleading and raising wrong expectations. It would be more appropriately titled 'TREM2-deficiency reduces the efficacy of immunotherapeutics amyloid uptake' and not clearance, since other aspects of clearance mechanisms were not addressed e.g.. degradation.

Referee #3 (Remarks):

The authors show that CRISPR/Cas9-induced loss of TREM2 significantly reduced uptake of pre-aggregated Aβ42 in N9 microglia cell lines, bone marrow derived macrophages derived from TREM2 knock-out mice, and primary microglia cells from TREM2 knock-out mice.

Compensatory increases in Syk phosphorylation may be a mechanism for TREM2 independent uptake of antibody-antigen complexes in TREM2 knock-out conditions.

In ex-vivo experiments with brain section derived from plaque-bearing transgenic mice, TREM2 deficiency in added macrophages resulted in reduced phagocytosis s of tissue amyloid plaques; this was partially compensatory by higher antibody concentrations suggesting the possibility that antibody-mediated immunotherapy may still be effective in clearing amyloid in patients with compromised TREM2 function.

1st Revision - authors' response 19 May 2016

Referee #1

(1) Did the authors look in their in vitro/ex vivo assays for the role of soluble TREM2, i.e. supplementation of knockout cells with soluble TREM2 from wt cells etc.? This would be very informative for understanding the role - if any - of this molecule in the overall scheme.

We addressed this question by adding increasing amounts of sTREM2 to the Ab uptake assays (up to 10 times more as compared to its physiological concentration in plasma of mice). However, sTREM2 did not rescue reduced uptake capacity of BMDM derived from the *Trem2* knockout. This is in line with the data demonstrating that full-length TREM2 together with its binding partner DAP12 trigger the signaling events for phagocytosis in a cell-autonomous manner. These data are now shown in the new Fig. 1I.

(2) Non-specialists would benefit from an even clearer discussion of the discrepancy with the TREM2 knockout mouse study reporting reduced pathology. Would the authors suggest that cross breeding a TREM2 KO mouse with a relatively mild Ab overproducer (e.g. TG2576) should lead to increased amyloid pathology at relatively early age (e.g. around 12 months)?

We are now discussing this issue as requested in the Discussion. (Page 11)

(3) I would rephrase the last sentence of the results suggesting that the data of the ex vivo assay indicate that TREM2 patients would benefit from amyloid immunotherapy, because the required

antibody concentration of the ex vivo assay would be reachable - the assay is after all contrived and does not reflect in vivo human microglia. I think it is fair to state that such patients would likely require higher antibody doses, but whether these doses are reachable in the clinic is hard to know and not critical for the main conclusion of the present study.

We have deleted the last sentence to avoid any overstatement. (Page 9)

Referee #2

Point 1

The mAb11 antibody was used, which has not been described in the manuscript.

In our manuscript we referred to Lathuilière A, et al. Brain 2016, which by the time of our submission was in press. The paper is now online available. In that paper the antibody is described in detail. Nevertheless, we now describe the antibody characteristics in detail in the Materials and Methods. (Page 14-15)

An 'irrelevant antibody 6687 raised to the C-terminus of APP was used as a negative control'. What does irrelevant mean here?

We specifically used a control antibody, which recognizes APP (its very C-terminus) but not Ab. We have explained that in more detail and the term "irrelevant" was eliminated to avoid confusion. (Page 5 & 14)

It would be more appropriate to include other antibodies, which recognize specific Abeta epitopes.

We have done that. In our manuscript we used two independent anti-Ab antibodies namely, 2D8 (Ab 1-16) and mAb11 (conformational epitope). As a negative control, we used the non-Aβ antibody 6687 raised to the APP C-terminus and appropriate isotype controls.

Also, A11, according to a statement given in the manuscript, has 'similar properties' to Gantenerumab, which is supposed to recognize fibrils as described in ref 36. Depending on what isoforms of Abeta and aggregate states A11 is recognizing it could explain one or the other result presented without TREM2 being involved, especially Western blot data shown in figure 4. There is no reference given where A11 has been characterized in detail, or one that may not rely on oligomer-fibril recognition.

We referred to Lathuilière A, et al. (Brain 2016), which by the time of our submission was *in press*. The paper is now online available and we describe the details of the antibody characteristics in the Materials and Methods. (Page 14-15)
mAb11 clearly stimulates amyloid plaque clearance. The removal of mAb11 covered amyloid plaques strongly indicates that all pathologically relevant Ab forms are detected. Moreover, the western blot in Fig. 4 is performed after full denaturation using even another independent antibody. Thus we detect the entire Ab population independent of its aggregation status. Even in the high molecular regions of the SDS-gel no unresolved aggregates were visible. (Source data for Fig. 4E)

Point 2
This manuscript is using quite artificial systems, based on the use of a fluorescently labeled Abeta42 that we are expected to supposedly exist as aggregates, then co-incubated with various antibodies, which is then applied to purified microglia or BMDM cultures, or glia co-cultured with brain slices.

We did not add Ab preparations to the brain slices. The slices were from 6-months old APP/PS1 mice containing *in vivo* produced amyloid plaques, which are well known to closely resemble pathological properties of human plaques. Their removal is greatly stimulated upon addition of anti-Ab antibodies. Thus this point must be a misunderstanding. Moreover, synthetic and naturally produced Ab aggregates were both efficiently engulfed after antibody stimulation demonstrating that the fluorescent Ab peptides used did not reflect an artificial system (see also next comment).

Does this fluorescent tag modify the aggregation behavior or binding of the antibodies? Are similar results obtained using non-fluorescently labeled Abeta42?

The validity of this approach was independently confirmed by clearance of amyloid plaques produced in the brain of transgenic "Alzheimer mice", which closely resembling human plaques. Moreover, there is a large literature describing that the fluorescent label does not change the aggregation behavior of Aß significantly (see for example Barrett JP. *et al*. 2015; Li, W. *et al*. 2013; Chakrabarty, P. *et al*. 2010; Fleisher-Berkovich *et al.* 2010; Hickman, SE. *et al.* 2008; Nazer, B. *et al.* 2008). Furthermore, the uptake assays and the plaque clearance assay clearly do not depend on certain aggregation stages of Ab, since specifically amyloid plaques contain all types of pathological relevant species and these are efficiently cleared after addition of anti-Ab antibodies.
Finally, our antibodies clearly bind fluorescently labeled Ab, otherwise they would not selectively trigger Ab uptake.

Is Abeta42 the only species of amyloid to play a role in toxicity/pathology of Alzheimer's disease?

This is not the scope of our manuscript. However, clearance of amyloid plaques shows that all pathologically relevant species are engulfed and degraded. (Fig. 4A, B and E)

Point 3

Microglia and BMDM were cultured with brain slices from APP/PS1 mice; however, no marker is used at any point to identify these cells like cell-specific markers, other than CD68.

The identity of microglia and BMDM was additionally estimated by flow cytometry using an anti CD11b antibody. This demonstrated more than 90% purity of the cell preparations. (See new Fig. EV1)

What regions of the brain were used to prepare slices?

We used sagittal frozen sections from one entire hemisphere of a brain for each experiment. This is now mentioned in the Materials and Methods as well as in figure legends (Page 16 & 25). We have also included a schematic figure showing the experimental set up (new Fig. 3A) to avoid any misunderstandings.

Furthermore, whether any resident microglia or BMDM present in the brain slices used, were not assessed prior to the co-culturing with exogenously added glia. Is there a contribution of these resident glial cells to the phagocytosis of Abeta-antibody complexes?

Since we used cryosections there are no viable cells from the tissue interfering with the analysis. Moreover, we included "no cell" control in Fig. 4.

Since TREM2 has been shown to affect microglia survival and these amyloid uptake assays on slices lasted 24 hours (post cell seeding), how can the authors ascertain that the seeded microglia are responsible for amyloid uptake? What is the survival of TREM2-/- microglia and BMDM?

In the context of the assays used we did not observe differences in cell number of wt versus *Trem2* ko microglia/BMDM as assessed by CD68 immunostaining after termination of the assay (i.e. time of quantification). This essential control experiment is shown in Fig. 4C & D of our manuscript.

Point 4

No point 4 is listed.

Point 5

Syk is postulated to be a downstream effector of TREM2.

Syk is not just "postulated" to be a downstream effector of TREM2, but very well established as shown by numerous publications (for review see: J. Klesney-Tait, I. R. Turnbull, M. Colonna, The TREM receptor family and signal integration. Nat. Immunol. 7, 1266–1273 (2006)).

In figure 2, the levels of phosphorylated Syk were normalized only with actin. Generally, one should determine the proportion of total Syk that was phosphorylated.

We have now normalized the pSyk data to total Syk as requested. This is now shown in the new Fig. 2E. Note that this did not change our conclusions.

Furthermore, this seems like a piece of hanging data. The role or contribution of Syk is not further explored in this antibody-mediated uptake of Abeta42.

To address this point, we added new data showing that *Trem2* knockout BMDM upregulate Fcg-receptors. As a consequence pSyk levels are further enhanced upon Ab-2D8 stimulation. These data are now shown in the new Fig. 2A, B & C. Upregulation of Fcg-receptors further supports a compensatory mechanism in the *Trem2* knockout cell.

Point 6
There are relevant details regarding experimental design that are missing. For example, in Figure 4, what marker was used to determine cell number? This is not found in the figure legend nor in the figure itself.

We apologize for the omission of this information in Fig. 4. CD68 was used as a marker. This is now shown in the figure and mentioned in the corresponding legend. (Page 25)

Other comments
The title of the manuscript is misleading and raising wrong expectations. It would be more appropriately titled 'TREM2-deficiency reduces the efficacy of immunotherapeutics amyloid uptake' and not clearance, since other aspects of clearance mechanisms were not addressed e.g.. degradation.

We can certainly change the title accordingly. However, our data using the *ex-vivo* model clearly show that amyloid plaques disappear, i.e. they are cleared. (Fig. 4A, B & E)

Referee #3
No critical points were raised.

2nd Editorial Decision 08 June 2016

Thank you for the submission of your revised manuscript to EMBO Molecular Medicine. We have now received the enclosed report from the referee who was asked to re-assess it. As you will see this

reviewer is now supportive and I am pleased to inform you that we will be able to accept your manuscript pending final editorial amendments.

***** Reviewer's comments *****

Referee #2 (Remarks):

All points raised have sufficiently been addressed.

EMBO PRESS

YOU MUST COMPLETE ALL CELLS WITH A PINK BACKGROUND ➔

PLEASE NOTE THAT THIS CHECKLIST WILL BE PUBLISHED ALONGSIDE YOUR PAPER

Corresponding Author Name: Prof. Dr. Dr. h.c. Christian Haass
Journal Submitted to: EMBO Molecular Medicine
Manuscript Number: EMM-2016-06370

Reporting Checklist For Life Sciences Articles (Rev. July 2015)

This checklist is used to ensure good reporting standards and to improve the reproducibility of published results. These guidelines are consistent with the Principles and Guidelines for Reporting Preclinical Research issued by the NIH in 2014. Please follow the journal's authorship guidelines in preparing your manuscript.

A- Figures
1. Data
The data shown in figures should satisfy the following conditions:

- ➔ the data were obtained and processed according to the field's best practice and are presented to reflect the results of the experiments in an accurate and unbiased manner.
- ➔ figure panels include only data points, measurements or observations that can be compared to each other in a scientifically meaningful way.
- ➔ graphs include clearly labeled error bars for independent experiments and sample sizes. Unless justified, error bars should not be shown for technical replicates.
- ➔ if n< 5, the individual data points from each experiment should be plotted and any statistical test employed should be justified
- ➔ Source Data should be included to report the data underlying graphs. Please follow the guidelines set out in the author ship guidelines on Data Presentation.

2. Captions
Each figure caption should contain the following information, for each panel where they are relevant:

- ➔ a specification of the experimental system investigated (eg cell line, species name).
- ➔ the assay(s) and method(s) used to carry out the reported observations and measurements
- ➔ an explicit mention of the biological and chemical entity(ies) that are being measured.
- ➔ an explicit mention of the biological and chemical entity(ies) that are altered/varied/perturbed in a controlled manner.

- ➔ the exact sample size (n) for each experimental group/condition, given as a number, not a range;
- ➔ a description of the sample collection allowing the reader to understand whether the samples represent technical or biological replicates (including how many animals, litters, cultures, etc.).
- ➔ a statement of how many times the experiment shown was independently replicated in the laboratory.
- ➔ definitions of statistical methods and measures:
  - common tests, such as t-test (please specify whether paired vs. unpaired), simple χ2 tests, Wilcoxon and Mann-Whitney tests, can be unambiguously identified by name only, but more complex techniques should be described in the methods section;
  - are tests one-sided or two-sided?
  - are there adjustments for multiple comparisons?
  - exact statistical test results, e.g., P values = x but not P values < x;
  - definition of 'center values' as median or average;
  - definition of error bars as s.d. or s.e.m.

Any descriptions too long for the figure legend should be included in the methods section and/or with the source data.

Please ensure that the answers to the following questions are reported in the manuscript itself. We encourage you to include a specific subsection in the methods section for statistics, reagents, animal models and human subjects.

In the pink boxes below, provide the page number(s) of the manuscript draft or figure legend(s) where the information can be located. Every question should be answered. If the question is not relevant to your research, please write NA (non applicable).

USEFUL LINKS FOR COMPLETING THIS FORM

http://www.antibodypedia.com
http://1degreebio.org
http://www.equator-network.org/reporting-guidelines/improving-bioscience-research-repo
http://grants.nih.gov/grants/olaw/olaw.htm
http://www.mrc.ac.uk/Ourresearch/Ethicsresearchguidance/Useofanimals/index.htm
http://ClinicalTrials.gov
http://www.consort-statement.org
http://www.consort-statement.org/checklists/view/32-consort/66-title
http://www.equator-network.org/reporting-guidelines/reporting-recommendations-for-tun
http://datadryad.org
http://figshare.com
http://www.ncbi.nlm.nih.gov/gap
http://www.ebi.ac.uk/ega
http://biomodels.net/
http://biomodels.net/miriam/
http://jjj.biochem.sun.ac.za
http://oba.od.nih.gov/biosecurity/biosecurity_documents.html
http://www.selectagents.gov/

B- Statistics and general methods

Please fill out these boxes ➔ (Do not worry if you cannot see all your text once you press return)

1.a. How was the sample size chosen to ensure adequate power to detect a pre-specified effect size?	The biochemical experiments showed only very little interexperimental variations, therefore a common number of 3-6 experiments was performed.Page 23-26
1.b. For animal studies, include a statement about sample size estimate even if no statistical methods were used.	N/A
2. Describe inclusion/exclusion criteria if samples or animals were excluded from the analysis. Were the criteria pre-established?	N/A
3. Were any steps taken to minimize the effects of subjective bias when allocating animals/samples to treatment (e.g. randomization procedure)? If yes, please describe.	N/A
For animal studies, include a statement about randomization even if no randomization was used.	N/A
4.a. Were any steps taken to minimize the effects of subjective bias during group allocation or/and when assessing results (e.g. blinding of the investigator)? If yes please describe.	N/A
4.b. For animal studies, include a statement about blinding even if no blinding was done	N/A
5. For every figure, are statistical tests justified as appropriate?	YES. All figures contain a description of the statistical test used. (Page 17, 23-26)
Do the data meet the assumptions of the tests (e.g., normal distribution)? Describe any methods used to assess it.	We assessed the distribution of the data in an histogram and the distribution was not skewed. Therefore, parametric tests were used.
Is there an estimate of variation within each group of data?	The 95% confidence intervals of all groups compared are showed in the graphs.
Is the variance similar between the groups that are being statistically compared?	The groups compared have a similar n. We assess the variance with a box-plot and they were similar between the compared groups. (Page 17, 23-26)

C- Reagents

6. To show that antibodies were profiled for use in the system under study (assay and species), provide a citation, catalog number and/or clone number, supplementary information or reference to an antibody validation profile. e.g., Antibodypedia (see link list at top right), 1DegreeBio (see link list at top right).	See paragraph Antibodies (Page 14-15) of the Materials and Methods section.
7. Identify the source of cell lines and report if they were recently authenticated (e.g., by STR profiling) and tested for mycoplasma contamination.	Sources of the cell lines were reported in manuscript and the cells were not authenticated recently, no mycoplasma contamination was detected by PCR analysis. (Page 12)

* for all hyperlinks, please see the table at the top right of the document

D- Animal Models

8. Report species, strain, gender, age of animals and genetic modification status where applicable. Please detail housing and husbandry conditions and the source of animals.	All animal experiments were performed in accordance to local animal handling laws. Housing conditions included standard pellet food and water provided *ad libitum*, 12-hour light-dark cycle at temperature of 22 °C with cage replacement once per week and regular health monitoring. APP\PS1 mice (Radde R et al. 2006) were generated by Jucker's group. *Trem2* knockout mice (Turnbull IR *et al.* 2006) were provied by Colonna's group. (Page 12)
9. For experiments involving live vertebrates, include a statement of compliance with ethical regulations and identify the committee(s) approving the experiments.	Page 12
10. We recommend consulting the ARRIVE guidelines (see link list at top right) (PLoS Biol. 8(6), e1000412, 2010) to ensure that other relevant aspects of animal studies are adequately reported. See author guidelines, under 'Reporting Guidelines'. See also: NIH (see link list at top right) and MRC (see link list at top right) recommendations. Please confirm compliance.	All animal experiments were performed in accordance to local animal handling laws. (Page 12)

E- Human Subjects

11. Identify the committee(s) approving the study protocol.	N/A
12. Include a statement confirming that informed consent was obtained from all subjects and that the experiments conformed to the principles set out in the WMA Declaration of Helsinki and the Department of Health and Human Services Belmont Report.	N/A
13. For publication of patient photos, include a statement confirming that consent to publish was obtained.	N/A
14. Report any restrictions on the availability (and/or on the use) of human data or samples.	N/A
15. Report the clinical trial registration number (at ClinicalTrials.gov or equivalent), where applicable.	N/A
16. For phase II and III randomized controlled trials, please refer to the CONSORT flow diagram (see link list at top right) and submit the CONSORT checklist (see link list at top right) with your submission. See author guidelines, under 'Reporting Guidelines'. Please confirm you have submitted this list.	N/A
17. For tumor marker prognostic studies, we recommend that you follow the REMARK reporting guidelines (see link list at top right). See author guidelines, under 'Reporting Guidelines'. Please confirm you have followed these guidelines.	N/A

F- Data Accessibility

18. Provide accession codes for deposited data. See author guidelines, under 'Data Deposition'.<br><br>Data deposition in a public repository is mandatory for:<br>a. Protein, DNA and RNA sequences<br>b. Macromolecular structures<br>c. Crystallographic data for small molecules<br>d. Functional genomics data<br>e. Proteomics and molecular interactions	N/A
19. Deposition is strongly recommended for any datasets that are central and integral to the study; please consider the journal's data policy. If no structured public repository exists for a given data type, we encourage the provision of datasets in the manuscript as a Supplementary Document (see author guidelines under 'Expanded View' or in unstructured repositories such as Dryad (see link list at top right) or Figshare (see link list at top right).	N/A
20. Access to human clinical and genomic datasets should be provided with as few restrictions as possible while respecting ethical obligations to the patients and relevant medical and legal issues. If practically possible and compatible with the individual consent agreement used in the study, such data should be deposited in one of the major public access-controlled repositories such as dbGAP (see link list at top right) or EGA (see link list at top right).	N/A
21. As far as possible, primary and referenced data should be formally cited in a Data Availability section. Please state whether you have included this section.<br><br>Examples:<br>**Primary Data**<br>Wetmore KM, Deutschbauer AM, Price MN, Arkin AP (2012). Comparison of gene expression and mutant fitness in Shewanella oneidensis MR-1. Gene Expression Omnibus GSE39462<br>**Referenced Data**<br>Huang J, Brown AF, Lei M (2012). Crystal structure of the TRBD domain of TERT and the CR4/5 of TR. Protein Data Bank 4O26<br>AP-MS analysis of human histone deacetylase interactions in CEM-T cells (2013). PRIDE PXD000208	N/A
22. Computational models that are central and integral to a study should be shared without restrictions and provided in a machine-readable form. The relevant accession numbers or links should be provided. When possible, standardized format (SBML, CellML) should be used instead of scripts (e.g. MATLAB). Authors are strongly encouraged to follow the MIRIAM guidelines (see link list at top right) and deposit their model in a public database such as Biomodels (see link list at top right) or JWS Online (see link list at top right). If computer source code is provided with the paper, it should be deposited in a public repository or included in supplementary information.	N/A

G- Dual use research of concern

23. Could your study fall under dual use research restrictions? Please check biosecurity documents (see link list at top right) and list of select agents and toxins (APHIS/CDC) (see link list at top right). According to our biosecurity guidelines, provide a statement only if it could.	N/A
